# Age-Dependent Decline in Common Femoral Artery Flow-Mediated Dilation and Wall Shear Stress in Healthy Subjects

**DOI:** 10.3390/life12122023

**Published:** 2022-12-04

**Authors:** Mariam Bapir, Gavrielle R. Untracht, Julie E. A. Hunt, John H. McVey, Jenny Harris, Simon S. Skene, Paola Campagnolo, Nikolaos Dikaios, Ana Rodriguez-Mateos, David D. Sampson, Danuta M. Sampson, Christian Heiss

**Affiliations:** 1 Department of Clinical and Experimental Medicine, School of Biosciences, Faculty of Health & Medical Sciences, University of Surrey, Guildford GU2 7XH, UK; 2 Optical + Biomedical Engineering Laboratory, School of Electrical, Electronic and Computer Engineering, The University of Western Australia, Perth 6009, Australia; 3 Surrey Biophotonics, Advanced Technology Institute, School of Physics and School of Biosciences and Medicine, University of Surrey, Guildford GU2 7XH, UK; 4 School of Biosciences, Faculty of Health & Medical Sciences, University of Surrey, Guildford GU2 7XH, UK; 5 Department of Biochemical Sciences, School of Biosciences, University of Surrey, Guildford GU2 7XH, UK; 6 School of Health Sciences, University of Surrey, Guildford GU2 7XH, UK; 7 Mathematics Research Center, Academy of Athens, 11855 Athens, Greece; 8 Department of Nutritional Sciences, School of Life Course and Population Sciences, Faculty of Life Science and Medicine, King’s College London, London SE1 9NH, UK; 9 Surrey Biophotonics, Centre for Vision, Speech and Signal Processing and School of Biosciences and Medicine, University of Surrey, Guildford GU2 7XH, UK; 10 Institute of Ophthalmology, University College London, London SE5 9RS, UK; 11 Surrey and Sussex NHS Healthcare Trust, Redhill RH1 5RH, UK

**Keywords:** flow-mediated dilation, vascular ageing, femoral artery

## Abstract

Femoral artery (FA) endothelial function is a promising biomarker of lower extremity vascular health for peripheral artery disease (PAD) prevention and treatment; however, the impact of age on FA endothelial function has not been reported in healthy adults. Therefore, we evaluated the reproducibility and acceptability of flow-mediated dilation (FMD) in the FA and brachial artery (BA) (*n* = 20) and performed cross-sectional FA- and BA-FMD measurements in healthy non-smokers aged 22–76 years (*n* = 50). FMD protocols demonstrated similar good reproducibility. Leg occlusion was deemed more uncomfortable than arm occlusion; thigh occlusion was less tolerated than forearm and calf occlusion. FA-FMD with calf occlusion was lower than BA-FMD (6.0 ± 1.1% vs 6.4 ± 1.3%, *p* = 0.030). Multivariate linear regression analysis indicated that age (−0.4%/decade) was a significant independent predictor of FA-FMD (*R^2^* = 0.35, *p* = 0.002). The age-dependent decline in FMD did not significantly differ between FA and BA (*p_interaction agexlocation_* = 0.388). In older participants, 40% of baseline FA wall shear stress (WSS) values were <5 dyne/cm^2^, which is regarded as pro-atherogenic. In conclusion, endothelial function declines similarly with age in the FA and the BA in healthy adults. The age-dependent FA enlargement results in a critical decrease in WSS that may explain part of the age-dependent predisposition for PAD.

## 1. Introduction

The burden of lower extremity peripheral artery disease (PAD) is growing globally [1]. The prevalence of PAD increases with age and is more frequent in people with diabetes [1,2]. There are currently no established susceptibility biomarkers of lower extremity vascular health for use in PAD prevention, predictive or response biomarkers to guide PAD treatment [3]. Considering the known pathophysiology of vascular disease, the key role of endothelial dysfunction, and the known heterogeneity of both across the vascular tree [4], the measurement of lower limb-specific endothelial function appears to be a promising approach to monitor PAD risk, and to support targeted prevention and causal treatment development. We have previously shown that endothelial function as measured by flow-mediated dilation (FMD) in the femoral artery (FA) is decreased as compared to the brachial artery (BA) not only in patients with manifest PAD [5], but already in people with diabetes who are at increased risk for PAD but do not present with flow-limiting stenoses of lower extremity arteries [6]. More importantly, we have demonstrated that both FA-FMD and BA-FMD can be improved by supplementation with cocoa flavanols for healthy participants with and without type 2 diabetes [6]. While it is known that BA endothelial function decreases with age [7,8], the impact of age on FA-FMD has not been specifically investigated in healthy adults. These values could be used as a basis for future reference values. Therefore, we evaluated in the current set of studies the reproducibility and acceptability (Study 1&2), and age-dependence of FA-FMD together with BA-FMD in healthy individuals over a wide age range (Study 3). We hypothesize that in healthy individuals FA-FMD decreases with age at a similar degree to BA-FMD.

## 2. Materials and Methods

We performed three studies. We first compared the reproducibility and acceptability of BA-FMD with two different FA-FMD protocols using thigh and calf occlusion in healthy young participants on the same day (*n* = 10). In the second study, we performed reproducibility measurements of BA- and FA-FMD (calf occlusion) in healthy middle-aged people on 2 different days (*n* = 10). Finally, we performed cross-sectional measurements of FA (calf occlusion) and BA-FMD in healthy non-smoking participants over a wide age range (*n* = 50). 

The study was conducted according to the guidelines of the Declaration of Helsinki. All procedures were approved by the University of Surrey Ethics Committee and written informed consent was obtained from all study participants. The study was registered on clinical trials.gov (NCT04897191).

### 2.1. Study Participants

Seventy-three individuals (2 × *n* = 10 reproducibility and *n* = 53 cross-section studies, respectively) were recruited consecutively for the three studies at the University of Surrey (January–May 2021) and measurements were performed at the Clinical Investigations Unit. Three subjects were removed from the cross-sectional study as they were smokers. All participants were self-reported apparently healthy adults with a BMI of 20–30 kg/m^2^. Exclusion criteria were clinical signs or symptoms of manifest cardiovascular disease (coronary artery disease (CAD)), PAD, cerebrovascular disease), diabetes mellitus, ankle brachial pressure index (ABPI) <0.9 or >1.4, heart rhythm other than sinus rhythm, symptoms of acute infection, and active vasoactive medication. When asked, none of the participants reported a past medical history of chronic disease, including autoimmune or chronic inflammatory disease.

### 2.2. Study Design and Protocol

*Reproducibility and acceptability study same day (Study 1)*: We measured BA-FMD and FA-FMD with a 5-min distal occlusion (right forearm, thigh, and calf) in the following sequence: FA-FMD (thigh), BA-FMD (forearm), FA-FMD (thigh), BA-FMD (forearm), FA-FMD (calf). There was at least a 30-min interval between measurements on the same limb to allow recovery. To assess the feasibility and reproducibility of the different measurements, after explaining the procedure and before the commencement of measurements, we asked the participants to rate the expected discomfort based on a visual analogue scale for qualitative pain assessment from 0 (no discomfort) to 10 (most severe discomfort). Immediately after the first FMD measurement at each location, the participants rated the actual discomfort they experienced using the same scale. Finally, to assess acceptability, participants were asked if the discomfort was severe enough to prevent them from participating in future studies.

*Reproducibility FA-FMD calf occlusion (Study 2):* To assess the reproducibility of calf occlusion, we measured BA-FMD and FA-FMD in the morning on two different days under fasting conditions.

*Cross-sectional study (Study 3)*: To assess the age-dependence of FA-FMD (calf occlusion) and compare it with BA-FMD, we performed FMD measurements on the FA and the BA. We chose calf occlusion over thigh occlusion for FA-FMD measurement as thigh occlusion was not well tolerated by study participants. Together with FMD measurements, blood flow was assessed. In addition, we measured blood pressure with a cuff on the upper arm. The perfusion pressure of the legs was measured on the ankle arteries with a cuff placed around the lower leg following a standard protocol [9], allowing ABPI calculation using brachial systolic blood pressure. 

### 2.3. FMD and Blood Flow Analysis

FMD was measured in the right common femoral artery first (FA-FMD) then in the right brachial artery (BA-FMD) using 5-min distal occlusion with a fitted cuff in each case and ultrasound (Vivid I, 12 MHz linear array, GE Healthcare, Chalfont St Giles, UK) in combination with a digital video frame grabber and a semi-automated analysis system (Brachial Analyzer, MIA, Iowa City, IA, USA). All measurements were conducted in the morning after an overnight fast. To assess FA-FMD, depending on the study (see above), the cuffs were either placed around the mid-thigh proximal to the knee (FA-FMD thigh occlusion) or calf just distal to the knee joint (FA-FMD calf occlusion). For BA-FMD the cuff was placed around the proximal forearm (BA-FMD). Briefly, the FA or BA, respectively, was scanned above the femur head, or proximal to the elbow longitudinally, to obtain a clear anterior and posterior vessel wall-lumen interface. In ultrasound duplex mode, the Doppler spectrum showing blood flow velocity over time was visualised in parallel with the B-mode scan. After optimization of the settings, a video clip of 10 s duration was recorded for baseline pre-occlusion arterial diameter analysis. Then an appropriately sized blood pressure cuff around the thigh, proximal calf or forearm was inflated to 200 mmHg for 5 min. Starting shortly before deflation of the cuff, the recording of a 2-min video of the continuous duplex scan was started [10]. FMD was calculated as maximum relative diastolic diameter increase during post-occlusive reactive hyperaemia (PORH) as compared to baseline diameter: (diameter_maximum_ − diameter_baseline_)/diameter_baseline_ * 100. Lower limb and forearm blood flow rates were calculated as π * mean radius ^2 ^* mean velocity at baseline and peak immediately after reperfusion. The ratio of peak and baseline limb blood flow rate was used to calculate flow reserve. Wall shear stress was calculated as 8 * *m* * mean flow velocity/mean diameter, where blood viscosity (*m*) was assumed to be constant at 0.035 dyne*s/cm^2^ [11]. Shear rate was calculated as 8 * mean flow velocity/mean diameter [10].

### 2.4. Blood Pressure, Pulse Wave Velocity and ABPI Measurements

Office blood pressure and pulse wave velocity were measured using a sphygmomanometer device (Arteriograph24, Tensiomed, Budapest, Hungary) at the upper right arm in a supine position after 10 min of supine rest in a quiet room with the arm resting at heart level. Three blood pressure measurements were taken. The first reading was discarded and the remaining two were used to calculate an average which was used for further analyses [12]. ABPI was measured following the standard protocol described in clinical practice guidelines for the diagnosis of PAD [9]. Briefly, we measured the systolic pressure of the dorsalis pedis and posterior tibial artery at the ankle level using Doppler with a cuff placed around the lower leg. The higher systolic value was divided by the average systolic pressure measured on the arm.

### 2.5. Statistical Analysis and Sample Size Calculation

The baseline characteristics of the study population are expressed as mean values ± standard deviation (SD). Reproducibility was expressed as the average deviations (AD) and standard deviations of individual differences in FMD measurements on two occasions. AD was calculated as the group mean of individual differences between repeated measurements (second measurement minus first measurement). Differences between FA and BA measurements were compared with repeated measurements ANOVA and paired sample t-tests. Expected and experienced pain VAS data were treated as ordinal scale non-parametric data and compared using the Wilcoxon test. Correlations were Pearson’s correlations. Multivariate linear regression analyses were performed to identify significant independent predictors of BA-FMD and FA-FMD (dependent variables). Analyses were computed with SPSS 28 (IBM, Armonk, NY, USA). Based on previous BA FMD studies, we expected a correlation coefficient (Pearson’s) between age and FMD of −0.4 [7]. With a correlation under the null hypothesis of 0.0, alpha 0.05, and power of 0.8 a sample size of at least 47 would be required, which aligns with the current n = 50 cohort.

## 3. Results

### 3.1. Reproducibility and Acceptability

**Figure 1:**
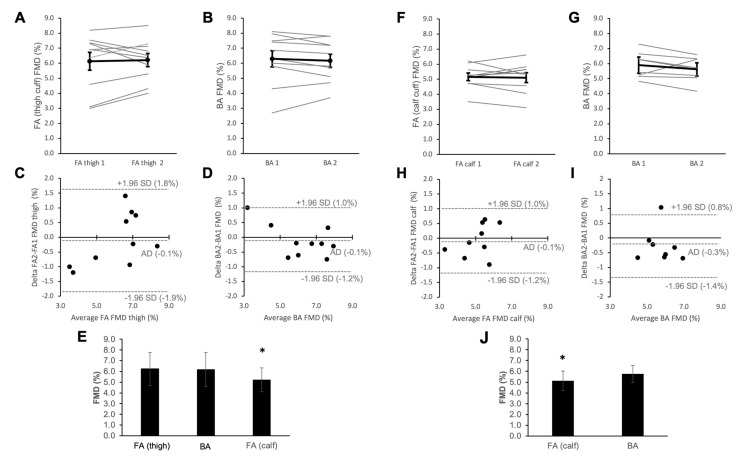
Reproducibility of flow-mediated dilation (FMD) in the common femoral artery (FA) with occlusion of the thigh and calf and brachial artery (BA). (**A**–**E**): Study 1 with consecutive measurement of FA-FMD (thigh), BA-FMD, FA-FMD (thigh), BA-FMD and FA-FMD (calf) in *n* = 10 healthy individuals (Table 1A). Acceptability is summarised in Figure 2. E: values are based on average of two measurements for BA-FMD and FA-FMD (thigh) for each individual shown in panel A&B and single value measured for FA-FMD (calf, individual points not shown). * *p* < 0.05 based on repeated-measurements ANOVA. (**F**–**J)**: Study 2 with measurement of BA-FMD and FA-FMD (calf) in *n* = 10 healthy individuals (Table 1B). J: values are based on average of 2 measurements for BA-FMD and FA-FMD (thigh) for each individual shown in panel F&G. * *p* < 0.05 based on paired t-test. A, B, F, **G**: Individual datapoints of *n* = 10 (grey lines) together with mean and SD. C, D, H, I: Bland–Altman plots based on values shown in A, B, F, and G, respectively. Dotted lines are average deviation (AD) of two measurements and 1.96 standard deviation (SD) above or below AD.

The characteristics of study participants are shown in Table 1A. Measurements of the BA-FMD and FA-FMD (thigh) were repeated twice in the same participants followed by a single FA-FMD (calf) measurement. The mean BA-FMD value of the first measurement was 6.3 ± 1.7% and the second was 6.2 ± 1.4% (mean ± SD, *p* = 0.486 (paired t-test)). The average deviation between the two BA-FMD measurements was −0.1% with a standard deviation of 0.6% (See Figure 1 also for Bland-Altman plots). The mean FA-FMD value (thigh occlusion) of the first measurement was 6.1 ± 1.4% and the second was 6.2 ± 1.1% (*p* = 0.776). The average deviation between both FA-FMD (thigh) measurements was −0.1% with a standard deviation of 0.9%. Whereas BA-FMD and FA-FMD (thigh occlusion) did not significantly differ (BA-FMD: 6.2 ± 1.5% vs FA-FMD (thigh): 6.2 ± 1.6%, *p* = 0.746), FA-FMD with calf occlusion (5.2 ± 1.1%) was significantly lower than BA-FMD (*p* = 0.008), and FA-FMD with thigh occlusion (*p* = 0.007, average difference: −1.0 ± 0.9%).

**Figure 2:**
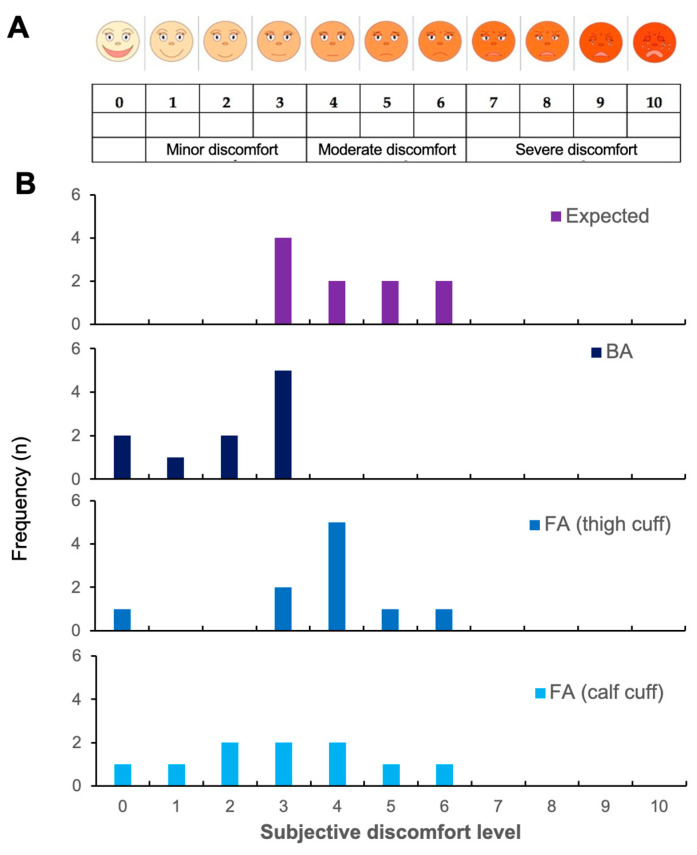
Discomfort level of FMD occlusion protocols. Shows participant reported expected discomfort based on a (**A**) visual analogue scale (**B**) before measurements (expected) and experienced pain after measurement of flow-mediated dilation (FMD) in brachial artery (BA) and femoral artery (FA) with 5-min occlusion with a cuff around the thigh and calf. Performed as part of Study 1 measurements.

In terms of acceptability, 4/10 anticipated minor and 6/10 moderate discomfort before the measurements (median 4 range 3–6) (Figure 2B). Post-measurement, all participants rated the discomfort as absent to minor (median 2.5, range 0–3, *p* = 0.004 vs. expected) for BA, whereas 4/10 indicated moderate discomfort for FA calf (median 3, range 0–6, *p* = 0.005 vs. expected) with FA thigh being least well tolerated with 7/10 rating their discomfort as moderate (median 4, range 0–6, *p* = 0.157 vs. expected, *p* = 0.038 vs. calf). None of the participants stated the discomfort was severe enough to prevent them from participating in future trials.

As thigh occlusion was less well tolerated and participants of Study 1 were by chance younger than people receiving FA-FMD in a clinical setting, we performed a second study to evaluate the reproducibility of FA-FMD (calf occlusion) as compared to BA-FMD in slightly older individuals on two different days (see characteristics in Table 1B). The mean BA-FMD value of the first measurement was 5.9 ± 0.9% and the second was 5.6 ± 0.8% (*p* = 0.221). The average deviation of both BA-FMD measurements was −0.3% with a standard deviation of 0.8%. The mean FA-FMD (calf occlusion) of the first measurement was 5.2 ± 0.8% and of the second was 5.1 ± 0.1% (*p* = 0.742). The average deviation of both FA-FMD measurements with calf occlusion was −0.1% with a standard deviation of 0.6%. Again, the FA-FMD values were significantly lower than BA-FMD (each average of both days, BA: 5.8 ± 0.8% vs FA (calf): 5.1 ± 0.9%, *p* = 0.036).

Due to the lower acceptability of thigh occlusion and equivalent or numerically better reproducibility of FA-FMD with calf occlusion, we chose the calf occlusion protocol for the cross-sectional study to evaluate the age-dependence of FA-FMD.

### 3.2. Age-Dependent Decline in FA-FMD and BA-FMD

In apparently healthy individuals aged 22-76 years (*n* = 50, Table 2), FA-FMD with calf cuff was significantly lower than BA-FMD (6.0 ± 1.1% vs 6.4 ± 1.3%, *p* = 0.030; individual average difference (delta) 0.5 ± 1.1%). In addition, the baseline diameter, mean flow velocity and flow rate were significantly higher in the FA as compared to the BA whereas the WSS and SR were significantly lower. At the onset of PORH, the flow rate was significantly higher, but mean flow velocity, WSS, SR and flow reserve were lower in the FA as compared to the BA.

**Figure 3:**
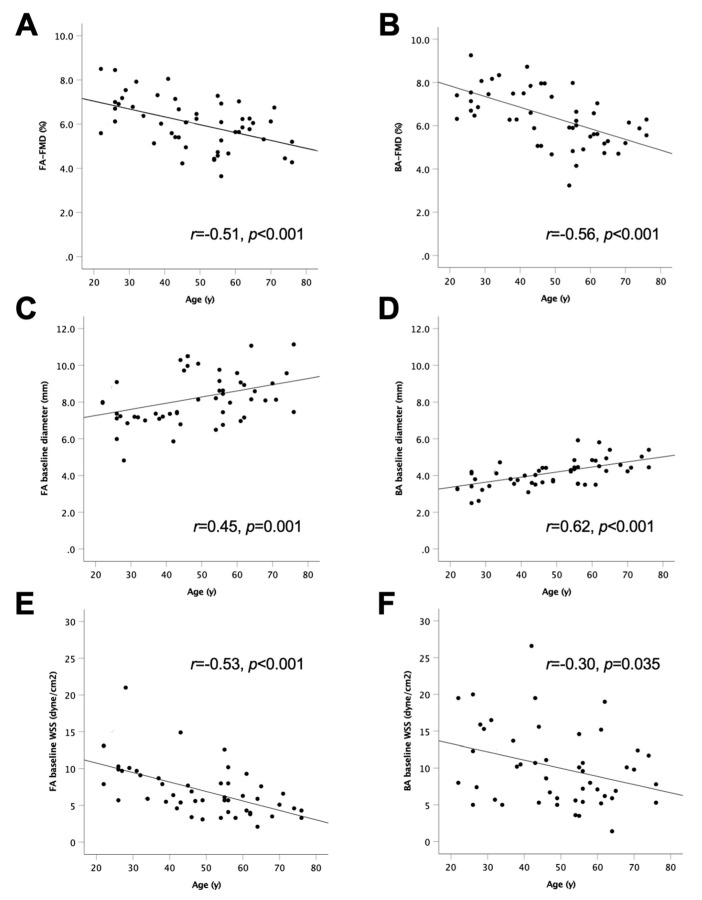
Scatter plots showing individual values of measurements on femoral artery (FA; **A**,**C**,**E**) and brachial artery (BA; **B**,**D**,**F**) in *n* = 50 healthy non-smoking participants. (**A**,**B**) are flow-mediated dilation (FMD) with calf occlusion, (**C**,**D**) baseline diameter, (**E**,**F**) wall shear stress (WSS). Note that based on repeated measurements ANOVA, none of the slopes of each measurement differed between FA and BA.

As summarised in Table 3, FA-FMD and BA-FMD correlated significantly (*r* = 0.61, *p* < 0.001). Both FA-FMD and BA-FMD correlated inversely with age and baseline diameter and positively with WSS (Figure 3). 

Multivariate linear regression analysis, including age, sex, BMI, baseline diameter and systolic blood pressure, explained 35% (adjusted *R*^2^ = 0.35, *p* = 0.002) of the variability of FA-FMD. In this model, only age was a significant independent predictor of FA-FMD. According to the unstandardised coefficient B in this model, with every decade FA-FMD was lower by 0.36% (*p* < 0.001). Similar results were seen in terms of BA-FMD (*adjusted R*^2^ = 0.43, *p* < 0.001) with age, sex, and baseline diameter being independent predictors. According to the unstandardised coefficient B, with every decade and every 1 mm larger BA, BA-FMD was lower by 0.41% (*p* = 0.002) and 0.67% (*p* = 0.042), respectively and female sex had 0.92% higher BA FMD than male (*p* = 0.025). Repeated measurements ANCOVA confirmed that age was a significant determinant of FMD decrease (*p_age_* < 0.001) and it also indicated that the age-dependent decrease of FMD did not significantly differ between FA and BA (*p_location_* = 0.140, *p_interaction locationxage_* = 0.388). It further indicated that the age-dependent increases in baseline diameters (*p_age_* < 0.001, *p_location_* = 0.428, *p_interaction locationxage_* = 0.389) and decreases in baseline WSS (*p_age_* < 0.001, *p_location_* = 0.394, *p_interaction locationxage_* = 0.779) did not differ significantly between FA and BA. While the peak PORH WSS was not quite statistically significantly related to age, there was also no difference between the effect of age on BA and FA values (*p_age_* = 0.058, *p_location_* = 0.022, *p_interaction locationxage_* = 0.605).

As WSS below 5 dyne/cm^2^ is regarded as pro-atherogenic [13], we evaluated the prevalence (baseline WSS) according to age. In the entire group (*n* = 50), 28% (*n* = 14) and 6% (*n* = 3) of participants had FA and BA baseline WSS values below 5 dyne/cm^2^. Of note, all participants with <5 dyne/cm^2^ were older than 40 years and among the participants older than 40 years (*n* = 35) the percentage of individuals presenting with WSS values below 5 dyne/cm^2^ was 40% (FA) and 9% (BA).

## 4. Discussion

The key findings of the current studies are that FMD measurements can be performed reproducibly in the common FA using both thigh and calf occlusion with calf occlusion being better tolerated. The FA-FMD decrease inversely correlates with age to a similar degree as BA-FMD in healthy individuals. WSS is on average lower in the FA as compared to the BA and decreases to a critical level with age more frequently in the FA than the BA. 

We have recently published healthy BA-FMD values to be used as reference values for cardiovascular health assessment [7]. Our current data align with and confirm these previous findings in apparently healthy non-smoking adults in terms of absolute values, rate of age-dependent decline in the brachial artery (−0.41%/decade), and importance of the baseline brachial artery diameter (−0.67%/mm). The novel finding of the current study is that in apparently healthy people the age-dependent decline in endothelial function extends to the femoral artery with a similar degree of dependence on age to that of the brachial artery. However, the absolute values observed in the femoral artery were approximately 0.4% lower than in the brachial artery. This may be explained by the fact that in the cross-sectional study we used calf occlusion instead of thigh occlusion to induce reactive hyperaemia. This is supported by the results of our first reproducibility study (Figure 1) in which BA-FMD and FA-FMD with thigh occlusion were not significantly different, while FA-FMD with calf occlusion was significantly lower. This has been observed previously in the arm where upper arm occlusion leads to larger FMD as compared to forearm occlusion in part due to larger tissue cross-sections being occluded and leading to a larger degree of reactive hyperaemia [14]. The method with thigh occlusion would have better aligned with our previous study in which we showed similar FMD values in BA and superficial femoral artery (SFA) using thigh occlusion in participants without PAD [5]. We however decided against thigh occlusion protocols as previously used by us [5] and others [15] as this caused significantly more discomfort and was less well tolerated by participants than calf occlusion.

Our data could be used as reference values allowing the identification of people at risk for PAD (susceptibility biomarker) and as an organ-specific biomarker of lower limb health to therapeutically target one component of the underlying cause of PAD: endothelial dysfunction (predictive or response biomarkers) [3]. Our previous data clearly show that, in patients with PAD, FA-FMD is decreased as compared to the BA even after angioplasty of focal flow-limiting lesions [5]. More recently, we have demonstrated that in people with type 2 diabetes, who are at increased risk of developing PAD [2], FA-FMD is more impaired than BA-FMD [6]. Furthermore, we and others have shown that not only BA-FMD but also FA-FMD is amenable to therapeutic interventions such as cocoa flavanols [6], green tea [16], running/exercise [17], interruption of prolonged sitting [18], and counterpulsation [19]. The characterisation of femoral artery ageing may therefore contribute to a better understanding of how ageing may facilitate atherosclerosis development in the lower extremity and much less so in the upper extremity. We observed that the age-dependent increase in arterial mean baseline diameter is accompanied by impairments in WSS. It was previously observed that the age-dependent decline in BA-FMD was related to the diameter [7,20]. Due to the larger diameter of the femoral artery as compared to the brachial, this results in a greater proportion of participants with WSS values of less than 5 dyne/cm^2^, which is accepted to be a proatherogenic condition [13,21,22]. In the current cohort, 40% of participants older than 40 years (none in the younger) had femoral artery WSS of less than 5 dyne/cm^2^. We cautiously speculate these people may be at increased risk of developing PAD and targeted interventions may be able to prevent this. 

The potential clinical relevance of the current data lies in the fact that the values can serve as starting healthy reference values to allow indicative classification of values measured in patients as normal or decreased. Decreased values may indicate that the person is at increased risk of developing PAD (susceptibility biomarker). The limb-specific effect of treatments, including management of standard modifiable cardiovascular risk factors (SMuRFs) [23] and novel approaches that improve vascular health beyond SMuRFs [24,25], could be ascertained and monitored. Improvement in FMD with subjects who benefit from interventions can be independent from attenuation of classical risk factors such as blood pressure [26].

### Limitations

Several limitations apply to the study, the results and the FMD method in general. In the current study, we did not determine lipids, glucose, and cardiovascular family history, and there is the possibility that some of the participants had dyslipidaemia, insulin resistance, or familial risk. Therefore, true healthy reference values may be slightly higher or have a smaller variation. Another limitation of the FMD method for both BA and FA in terms of wider clinical adoption of the techniques lies in the fact that it only captures one time point, is time consuming, and requires high-quality ultrasound equipment, analysis software, and trained staff to perform the measurements [10]. Whether FMD measurements are cost-effective is not known [27]. The potential solution to this issue lies in the future development of personalized devices that can automatically and repeatedly register flow-mediated dilation [28] or endothelial function without the need for an FMD measurement as currently required. 

## 5. Conclusions

In healthy adults, endothelial function declines with age in the FA to a similar degree as in the BA. However, the age-dependent enlargement of the FA leads to a critical decrease in WSS that may explain part of the age-dependent predisposition for PAD. Taken together, the data provide a sufficiently strong proof-of-concept to warrant further studies in larger populations. Future studies would support generalisability of results, further characterise determinants of FA-FMD and validate FA-FMD as a limb-specific cardiovascular biomarker to either predict PAD development or limb prognosis. They would also enable establishing if an improvement in FA-FMD by interventions can predict prevention of PAD development or amputations.

## Figures and Tables

**Table 1:** Baseline characteristics of study population (A) reproducibility and acceptability (Study 1) and (B) FA-FMD calf occlusion reproducibility (Study 2). Values are mean and standard deviation (BMI = body mass index).

A	*n* (m/f)	2/8
	Age	28 ± 7
	Height (m)	1.68 ± 0.10
	Weight (kg)	72.0 ± 15.0
	BMI (kg/m^2^)	25.5 ± 5.6
	Smoker (n)	5
	Systolic blood pressure (mmHg)	118.0 ± 2.3
	Diastolic blood pressure (mmHg)	75.0 ± 1.9
B	*n* (m/f)	4/6
	Age	53 ± 10
	Height (m)	1.73 ± 0.11
	Weight (kg)	70.5 ± 12.7
	BMI (kg/m^2^)	23.6 ± 3.9
	Smoker (n)	0
	Systolic blood pressure (mmHg)	114.7 ± 11.3
	Diastolic blood pressure (mmHg)	73.3 ± 9.8

**Table 2:** Baseline (A) demographic and (B) vascular characteristics of cross-sectional study population. Values are mean and standard deviation and *p* values are from paired *t*-tests. (FMD = flow-mediated dilation, FA = femoral artery, BA = brachial artery, BMI = body mass index, WSS = wall shear stress, SR = shear rate, PORH = post-occlusive reactive hyperaemia).

A	*n* (m/f)	23/27		
	Age	48 ± 15		
	Height (m)	1.73 ± 0.10		
	Weight (kg)	70.0 ±12.2		
	BMI (kg/m^2^)	24.0 ± 3.4		
	Smoker (n)	0		
	Systolic blood pressure (mmHg)	118.5 ± 14.7		
	Diastolic blood pressure (mmHg)	71.0 ± 10.5		
	Ankle brachial pressure index	0.99 ± 0.09		
B		FA	BA	*p*
	FMD (%)	6.0 ± 1.1	6.4 ± 1.3	0.030
	Baseline diameter (mm)	8.11 ± 1.36	4.12 ± 0.74	<0.001
	Baseline mean flow velocity (cm/s)	19.4 ± 7.9	14.4 ± 6.4	<0.001
	Baseline flow rate (mL/min)	607 ± 296	119 ± 61	<0.001
	Baseline WSS (dyne/cm^2^)	7.0 ± 3.5	10.2 ± 5.3	<0.001
	Baseline SR (/s)	20.0 ± 10.1	29.0 ± 15.1	<0.001
	PORH mean flow velocity (cm/s)	49.7 ± 17.0	71.2 ± 25.4	<0.001
	PORH flow rate (mL/min)	1703 ± 658	689 ± 365	<0.001
	PORH WSS (dyne/cm^2^)	18.1 ± 8.4	48.9 ± 18.7	<0.001
	PORH SR (/s)	51.7 ± 24.1	139.8 ± 53.4	<0.001
	PORH flow reserve	3.1 ± 1.1	7.2 ± 7.0	<0.001

**Table 3:** Univariate Pearson correlations. r is Pearson’s correlation coefficient, values in bold indicate statistically significant correlations (BA = brachial artery, BMI = body mass index, DBP = diastolic blood pressure, FA = femoral artery, FMD = flow-mediated dilation, SBP = systolic blood pressure, WSS = wall shear stress).

	FA-FMD	BA-FMD	Age	Height	Weight	BMI	SBP	DBP	FA Baseline Diameter	FA Baseline Flow Rate	FA Baseline WSS	BA Baseline Diameter	BA Baseline Flow Rate
BA-FMD	***r* = 0.61,** ***p* < 0.001**												
Age	***r* = −0.51,** ***p* < 0.001**	***r* = −0.56,** ***p* < 0.001**											
Height	*r* = −0.04, *p* = 0.815	*r* = 0.17, *p* = 0.273	*r* = 0.06, *p* = 0.719										
Weight	*r* = −0.05, *p* = 0.745	*r* = −0.06, *p* = 0.700	*r* = 0.13, *p* = 0.394	***r* = 0.58,** ***p* < 0.001**									
BMI	*r* = −0.01, *p* = 0.932	*r* = −0.18, *p* = 0.217	*r* = 0.20, *p* = 0.154	*r* = −0.01, *p* = 0.931	***r* = 0.79,** ***p* < 0.001**								
SBP	*r* = −0.13, *p* = 0.379	*r* = −0.02, *p* = 0.894	***r* = 0.29,** ***p* = 0.042**	***r* = 0.34,** ***p* = 0.024**	***r* = 0.51,** ***p* < 0.001**	***r* = 0.39,** ***p* = 0.005**							
DBP	*r* = −0.27, *p* = 0.055	*r* = −0.06, *p* = 0.691	***r* = 0.30,** ***p* = 0.032**	***r* = 0.37,** ***p* = 0.012**	***r* = 0.38,** ***p* = 0.010**	*r* = 0.22, *p* = 0.132	***r* = 0.80,** ***p* < 0.001**						
FA baseline diameter	***r* = −0.40,** ***p* = 0.004**	***r* = −0.30,** ***p* = 0.032**	***r* = 0.45,** ***p* = 0.001**	***r* = 0.42,** ***p* = 0.004**	*r* = 0.27, *p* = 0.153	*r* = −0.05, *p* = 0.709	*r* = 0.27, *p* = 0.055	*r* = 0.24, *p* = 0.099					
FA baseline flow rate	*r* = −0.06, *p* = 0.666	*r* = 0.08, *p* = 0.568	*r* = −0.02, *p* = 0.871	***r* = 0.37,** ***p* = 0.015**	*r* = −0.08, *p* = 0.629	*r* = −0.22, *p* = 0.129	*r* = 0.14, *p* = 0.330	*r* = 0.17, *p* = 0.234	***r* = 0.51,** ***p* < 0.001**				
FA baseline WSS	***r* = 0.38,** ***p* = 0.008**	***r* = 0.32,** ***p* = 0.026**	***r* = −0.53,** ***p* < 0.001**	*r* = −0.13, *p* = 0.418	*r* = −0.26, *p* = 0.098	*r* = −0.13, *p* = 0.373	*r* = −0.10, *p* = 0.484	*r* = −0.06, *p* = 0.695	***r* = −0.54,** ***p* < 0.001**	***r* = 0.32,** ***p* = 0.024**			
BA baseline diameter	***r* = −0.43,** ***p* = 0.002**	***r* = −0.42,** ***p* = 0.003**	***r* = 0.62,** ***p* < 0.001**	***r* = 0.40,** ***p* = 0.006**	***r* = 0.39,** ***p* = 0.008**	***r* = 0.29,** ***p* = 0.042**	***r* = 0.34,** ***p* = 0.014**	***r* = 0.35,** ***p* = 0.012**	***r* = 0.43,** ***p* = 0.002**	*r* = 0.18, *p* = 0.217	***r* = −0.37,** ***p* = 0.010**		
BA baseline flow rate	*r* = −0.27, *p* = 0.062	*r* = 0.05, *p* = 0.727	***r* = 0.37,** ***p* = 0.010**	***r* = 0.47,** ***p* = 0.001**	***r* = 0.41,** ***p* = 0.006**	*r* = 0.21, *p* = 0.150	***r* = 0.28,** ***p* = 0.050**	***r* = 0.34,** ***p* = 0.016**	*r* = 0.26, *p* = 0.078	*r* = 0.26, *p* = 0.081	*r* = −0.14, *p* = 0.339	***r* = 0.59,** ***p* < 0.001**	
BA baseline WSS	*r* = 0.09, *p* = 0.539	***r* = 0.51,** ***p* < 0.001**	***r* = −0.30,** ***p* = 0.035**	*r* = −0.01, *p* = 0.953	*r* = 0.06, *p* = 0.681	*r* = −0.11, *p* = 0.472	*r* = −0.09, *p* = 0.550	*r* = 0.07, *p* = 0.611	*r* = −0.26, *p* = 0.073	*r* = 0.00, *p* = 0.987	*r* = 0.24, *p* = 0.098	***r* = −0.41,** ***p* = 0.004**	***r* = 0.40,** ***p* = 0.005**

## Data Availability

Data are available upon reasonable request to the corresponding author.
